# Bone marrow transplantation corrects haemolytic anaemia in a novel ENU mutagenesis mouse model of TPI deficiency

**DOI:** 10.1242/dmm.034678

**Published:** 2018-05-21

**Authors:** Ashlee J. Conway, Fiona C. Brown, Elinor J. Hortle, Gaetan Burgio, Simon J. Foote, Craig J. Morton, Stephen M. Jane, David J. Curtis

**Affiliations:** 1 Australian Centre for Blood Diseases, Central Clinical School, Monash University, Melbourne 3004, Australia; 2 The John Curtin School of Medical Research, Australian National University, Canberra 0200, Australia; 3 Australian Cancer Research Foundation Rational Drug Discovery Centre, St. Vincent's Institute of Medical Research, Fitzroy 3065, Australia; 4 The Alfred Hospital, Melbourne 3004, Australia; 5 Central Clinical School, Monash University, Melbourne 3004, Australia

**Keywords:** Erythropoiesis, N-ethyl-N-nitrosourea, Anaemia, TPI deficiency, Transplantation

## Abstract

In this study, we performed a genome-wide N-ethyl-N-nitrosourea (ENU) mutagenesis screen in mice to identify novel genes or alleles that regulate erythropoiesis. Here, we describe a recessive mouse strain, called RBC19, harbouring a point mutation within the housekeeping gene, *Tpi1*, which encodes the glycolysis enzyme, triosephosphate isomerase (TPI). A serine in place of a phenylalanine at amino acid 57 severely diminishes enzyme activity in red blood cells and other tissues, resulting in a macrocytic haemolytic phenotype in homozygous mice, which closely resembles human TPI deficiency. A rescue study was performed using bone marrow transplantation of wild-type donor cells, which restored all haematological parameters and increased red blood cell enzyme function to wild-type levels after 7 weeks. This is the first study performed in a mammalian model of TPI deficiency, demonstrating that the haematological phenotype can be rescued.

## Abstract (precis)

**Summary:** In a novel ENU mutagenesis mouse model of TPI deficiency, bone marrow transplantation was conducted to demonstrate that haemolytic and red blood cell glycolytic defects can be effectively rescued.

## INTRODUCTION

Triosephosphate isomerase (TPI, or TIM) is an anaerobic glycolysis enzyme encoded by the *TPI1* gene at chromosomal position 12p13 in humans (Ralser et al., 2008). It is responsible for the reversible conversion of dihydroxyacetone phosphate (DHAP) to D-glyceraldehyde 3-phosphate (GAP) and is ubiquitously expressed in all cells (Orosz et al., 2009; Orosz et al., 2006). Homozygous or compound heterozygous mutations within the *TPI1* gene result in the rare enzymopathy, TPI deficiency, which presents clinically with chronic haemolytic anaemia, cardiomyopathy, increased susceptibility to infections, neurodegeneration and death in early childhood (Sarper et al., 2013; Rosa et al., 1985; Fermo et al., 2010). Of the various glycolytic enzymopathies, TPI deficiency is considered the most severe in clinical presentation.

Of the ∼13 pathological *TPI1* mutations identified in humans so far, the Glu104Asp substitution has been the most commonly identified variant, believed to be responsible for up to 80% of all reported cases of TPI deficiency (Arya et al., 1997; Schneider and Cohen-Solal, 1996). This mutation is believed to cause impaired dimerisation of the enzyme, which is crucial for its catalytic activity, while other variants can also impair substrate binding to the active site (Ralser et al., 2006; Mainfroid et al., 1996; Oliver and Timson, 2017). Heterozygous carriers appear asymptomatic and maintain at least 50% TPI activity in all cells, while the loss of enzyme function in the erythrocytes, platelets or lymphocytes of homozygotes is more profound and ranges between zero and 30% of that of normal activity (Valentin et al., 2000; Hollán et al., 1993). Congenital mutations within glycolytic enzymes, such as TPI, have a particularly detrimental effect on the lifespan and function of red blood cells, which rely exclusively on anaerobic glycolysis for ATP production (Van Wijk and van Solinge, 2005; McMullin, 1999). Nonspherical haemolytic anaemia therefore manifests in all patients with TPI deficiency as a result of rapid red blood cell exhaustion and turnover, and poses a significant burden to health (Clay et al., 1982). Currently, no treatment is available nor has been trialled for TPI deficiency, likely owing to its rarity and fast mortality.

Models resembling TPI deficiency in experimental animals have been established previously. The TPI*^sugarkill^* (*sgk*; *Tpi^sgk-1^*) *Drosophila*, generated through ethyl methanesulfonate (EMS) mutagenesis, demonstrated reduced enzyme function and central brain pathology contributing to locomotive degeneration; a phenotype vaguely analogous to the neurodegenerative traits of human TPI deficiency (Palladino et al., 2002; Celotto et al., 2006). In a mammalian system, the *Tpi^a-m6Neu^* chemical mutagenesis mutant was the first viable homozygous mouse model, which presented with reduced enzyme activity in multiple tissues and evidence of haemolytic anaemia (Pretsch, 2009). While rescue studies in the *sgk* mutant were partially successful in restoring fly lifespan, locomotion and glycolytic output (Hrizo and Palladino, 2010), attempts to rescue the haematological phenotype in a mammalian model have not yet been trialled. Here, we describe a novel homozygous mouse model of TPI deficiency, called RBC19, which was generated in a genome-wide N-ethyl-N-nitrosourea (ENU) mutagenesis screen for defects in erythropoiesis. Whole-exome sequencing (WES) identified a missense mutation resulting in a phenylalanine-to-serine substitution at amino acid 57 of exon 1 (F57S). RBC19 homozygotes were viable and fertile, and presented with a macrocytic haemolytic anaemia, which closely paralleled the human disease. A bone marrow transplant was performed using wild-type donor cells to correct the haemolytic phenotype in RBC19 mice, restoring all red blood cell parameters and erythrocyte glycolytic function. This investigation represents the first rescue study of TPI deficiency in a mammalian model.

## RESULTS

### Characterisation of a homozygous mouse strain with macrocytic haemolytic anaemia

In an ENU mutagenesis screen designed to identify novel genes or alleles regulating erythropoiesis, we identified a mouse with macrocytic red blood cells, named RBC19. When crossed with wild-type (SJL) mice, ∼50% of progeny displayed macrocytosis and increased reticulocytes, indicating that the phenotype was fully penetrant. Mating two macrocytic mutants generated a third cohort of progeny at a ratio of ∼25% (data not shown). This presumed homozygous (m/m) population had more profound macrocytosis, a reduced red blood cell count and elevated reticulocytes (14.6%), compared with the heterozygous mice (Table 1). White blood cell and platelet indices were normal in all cohorts.

**Table 1.**
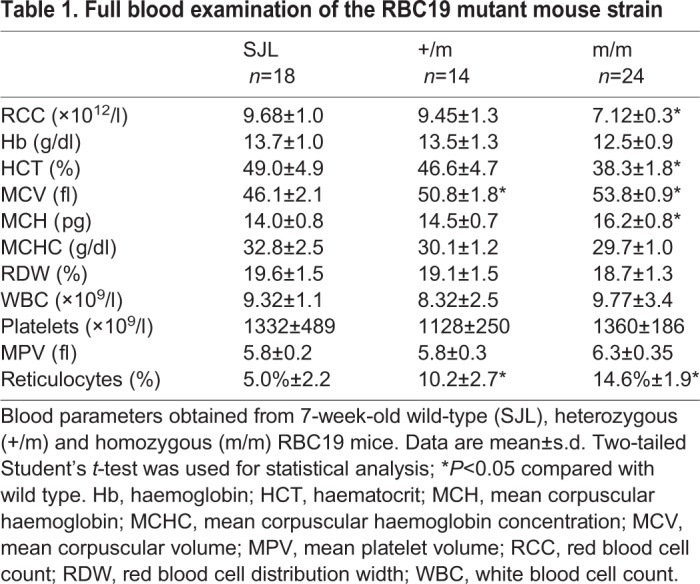
**Full blood examination of the RBC19 mutant mouse strain**

**Fig. 1.**
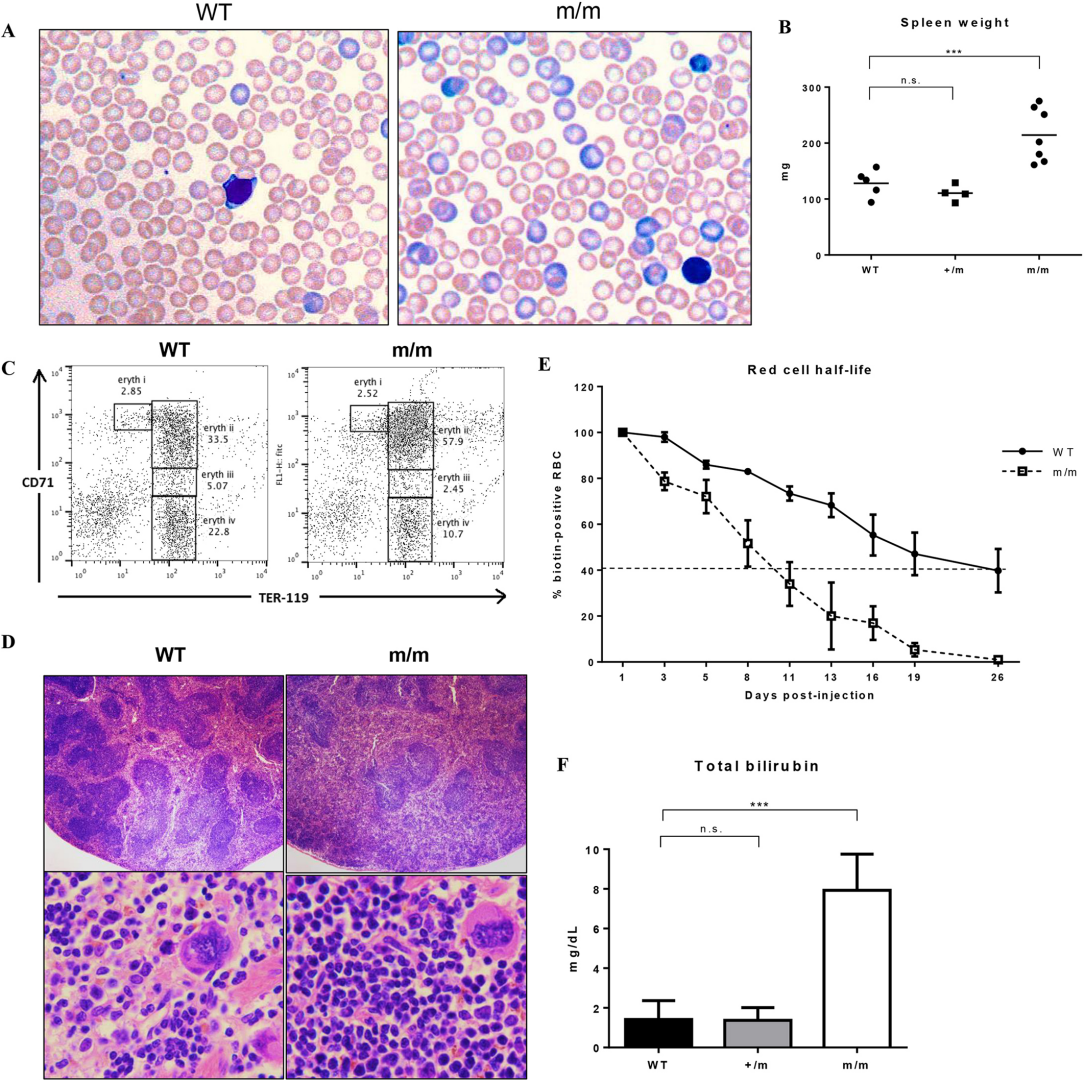
**Phenotype of the RBC19 mutant mouse.** (A) Peripheral blood smears of 7-week-old wild-type (WT) and RBC19 homozygous (m/m) mice, stained with May-Grünwald Giemsa. Macrocytic red blood cells and a high degree of polychromasia (blue) are seen in the homozygous film. (B) Wet spleen weights of wild-type (WT), heterozygous (+/m), and homozygous (m/m) mice. Individual values are shown, along with the mean (solid line). (C) Fluorescence-activated cell sorting (FACS) plot of live spleen cells stained with Ter-119 and CD71. There is a significant increase in early Ter-119^+^ CD71^hi^ cells (eryth ii) in the homozygotes. eryth i, proerythroblasts; eryth ii, basophilic erythroblasts; eryth iii, chromatophilic erythroblasts; eryth iv, orthochromic normoblasts. (D) Hematoxylin and Eosin (H&E) staining of sectioned spleens harvested from 2-month-old WT and m/m mice, photographed at low (×40) and high (×200) magnification. (E) Red blood cell half-life measured *in vivo* using biotinylation, showing a more rapid rate of red blood cell turnover in the homozygotes; *n*=4. Error bars represent s.d. (F) Total serum bilirubin quantification of WT, +/m and m/m mice. Data are mean±s.d.; *n*=6. ***P<0.0001, n.s., not significant.

Peripheral blood smears of RBC19 homozygotes (m/m) revealed a homogenous population of macrocytic red blood cells with mild pallor but normal shape (Fig. 1A). Significant polychromasia was visible in the blood smear, consistent with the elevated reticulocyte numbers. Increased spleen size was seen in homozygous, but not heterozygous, mice (Fig. 1B). Flow cytometric analysis of spleen cells identified a marked expansion of early Ter-119^+^ CD71^hi^ (Ly76^+^ Tfrc^hi^; *hi*, high) erythroblasts in the homozygous cohort, consistent with stress erythropoiesis (Fig. 1C). Histological sections of spleens revealed a dense, crowded red pulp packed with proliferating erythroid precursors (Fig. 1D). Biotinylation was employed to measure *in vivo* red blood cell half-life, as previously described (Conway et al., 2017), which demonstrated a significantly reduced half-life of RBC19 homozygous red blood cells (8 days), compared with wild-type red blood cells (17 days) (Fig. 1E). Total serum bilirubin was also markedly elevated in homozygotes compared with wild-type controls, but normal in the heterozygotes (Fig. 1F). Taken together, the results indicated that the RBC19 homozygous mutant mouse presented with a macrocytic red blood cell phenotype, with evidence of haemolytic anaemia.

### RBC19 mice harbour a missense mutation in the glycolysis enzyme gene, *Tpi1*

**Fig. 2.**
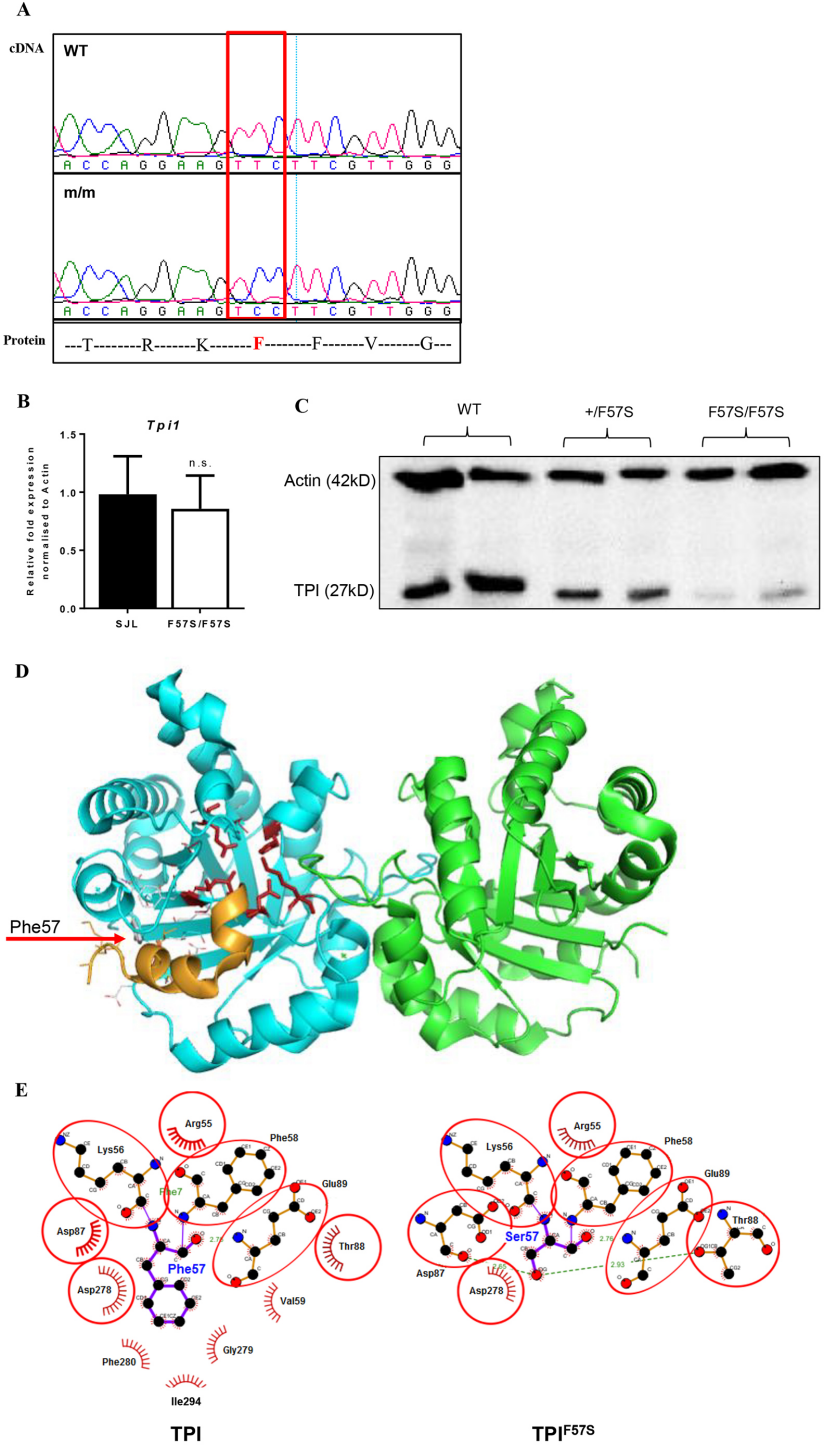
**Characterisation of the *F57S* mutation.** (A) Sanger sequencing of RBC19 homozygous cDNA shows a T→C substitution in *Tpi1*, resulting in a phenylalanine (F) to serine (S) amino acid change. (B) Quantitative RT-PCR for relative *Tpi1* mRNA expression in the bone marrow of wild-type (WT) and homozygous (F57S/F57S) mice. Data are mean±s.d.; *n*=4. (C) Western blot of the TPI protein from bone marrow lysates of WT, heterozygous (+/F57S) and homozygous (F57S/F57S) mice. (D) 3D protein modelling of the murine TPI dimer in ribbons (blue and green), showing the enzyme active site (red sticks). The location of F57 is highlighted (red arrow to grey stick), as well as its adjacent binding helix (orange ribbon). (E) 2D contact map of the F57 site and its surrounding amino acids in both the WT (TPI) and mutant (TPI^F57S^) forms. In the mutant, the F57S substitution is predicted to distort surrounding amino acid interactions and subsequently induce conformational changes to the protein.

The ENU-induced mutation was identified using WES, as previously described (Brown et al., 2013). Two samples of genomic DNA extracted from presumed RBC19 homozygotes, based on high mean corpuscular volume (MCV), were subjected to massively paralleled sequencing using the Illumina HiSeq platform. Reads were assembled against the SJL genomic sequence and subsequent bioinformatics identified a T→C base-pair mutation at position c170 in the first exon of *Tpi1*. This resulted in a predicted phenylalanine-to-serine substitution at amino acid 57 (F57S). Sanger sequencing of complementary DNA (cDNA) generated from the bone marrow of RBC19 homozygous mice (herein referred to as *Tpi1^F57S/F57S^*) confirmed the presence of the base-pair mutation (Fig. 2A). Quantitative real-time polymerase chain reaction (RT-PCR), performed on bone marrow, showed no significant differences in *Tpi1* mRNA expression levels between *Tpi1^F57S/F57S^* and wild-type mice (Fig. 2B); however, western blots revealed a significant reduction of TPI protein in the bone marrow lysates of homozygotes compared with wild-type samples, while heterozygous cells showed a ∼50% reduction in protein levels (Fig. 2C). This suggested that the F57S mutation had a detrimental effect on protein stability.

Computer modelling was used to help predict how the F57S mutation would affect TPI enzyme conformation and activity. In a three-dimensional (3D) model of the dimer's crystal structure, the F57 side chain was shown to be buried in a hydrophobic pocket, suggesting that it had an important role in stabilisation of the TIM ‘barrel’ configuration of the protein (Fig. 2D). In a contact map of the mutation site, it was further demonstrated that F57 played a central role in amino acid interactions via the packing of its aromatic side chain (Fig. 2E). Replacing this hydrophobic amino acid with a polar, hydrophilic amino acid (serine) is expected to disrupt those interactions, resulting in a misfolded protein and degradation. This likely explained the normal expression of *Tpi1* mRNA but markedly reduced levels of TPI protein.

### *Tpi1^F57S/F57S^* mice display haematological parallels to TPI deficiency

**Fig. 3.**
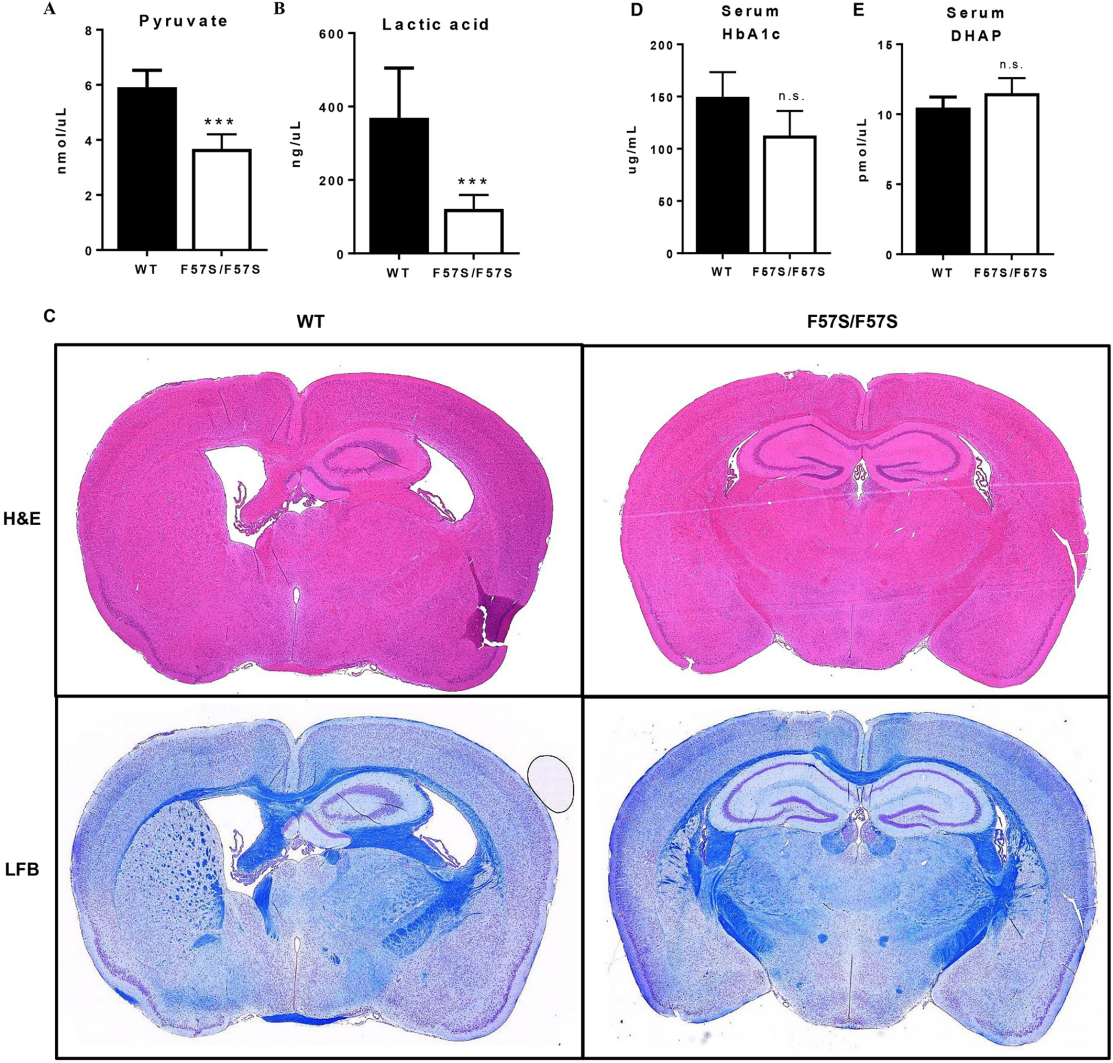
***Tpi1^F57S/F57S^* mice display a TPI deficiency-like phenotype without neuropathy.** (A,B) Pyruvate (A) and lactic acid (B) measured in the serum of wild-type (WT) and homozygous (F57S/F57S) mice. Data are mean±s.d.; *n*=6. (C) Sectioned brains of 9.5-month-old WT and F57S/F57S mice, stained with H&E (upper) and Luxol Fast Blue (LFB) (lower). (D,E) DHAP (D) and HbA1c (E) measured in the serum of WT and F57S/F57S mice. Data are mean±s.d.; *n*=5. ***P<0.0001, n.s., not significant.

Reduced erythrocyte TPI enzyme activity, in combination with nonspherical haemolytic anaemia, is a key diagnostic indicator of TPI deficiency. To examine the parallels between the *Tpi1^F57S/F57S^* mouse strain and the human condition, TPI enzyme function was analysed in lysates made of peripheral red blood cells and various other tissues (Table 2). In the homozygous population, the lowest enzyme activity was seen in red blood cells (10% of that of wild type), followed by brain (12%), bone marrow-derived macrophages (BMMs) (13%), and unsorted bone marrow (16%). Heterozygotes, which lacked a haemolytic phenotype, had a partial loss of enzyme activity in red blood cells (53% of that of wild type) and bone marrow cells (77%). Additionally, the end products of glycolysis, pyruvate (Fig. 3A) and lactic acid (Fig. 3B), were significantly reduced in the serum of *Tpi1^F57S/F57S^* mice compared with wild types, demonstrating the detrimental effect of the loss of TPI activity on the glycolysis pathway. Together, this indicated that the *Tpi1^F57S/F57S^* mutant is a novel model of TPI deficiency, and mimics the haematological and molecular defects typically observed in humans. Heterozygotes represented asymptomatic carriers.

**Table 2.**
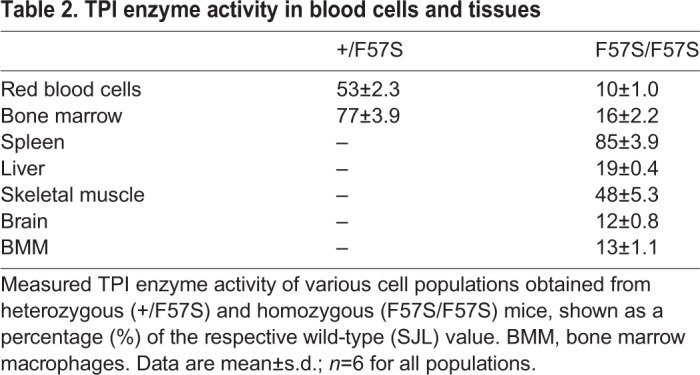
**TPI enzyme activity in blood cells and tissues**

No neurological phenotype was identified in the *Tpi1^F57S/F57S^* strain. Mice showed no loss of mobility or grip strength with age (data not shown), and histology of the brain did not reveal any pathological changes in 9-month-old mice (Fig. 3C). Serological tests of glycolytic metabolites typically identified in TPI-deficient patients with neurodegenerative symptoms, such as DHAP (Fig. 3D) and HbA1c – as an indirect measurement of methylglyoxal (Fig. 3E) – were also normal compared with wild-type serum values. These data suggest that the F57S mutation is not a catalyst of the neurodegenerative phenotypes often associated with TPI deficiency.

### Bone marrow transplantation rescues red blood cell defects in *Tpi1^F57S/F57S^* mice

**Fig. 4.**
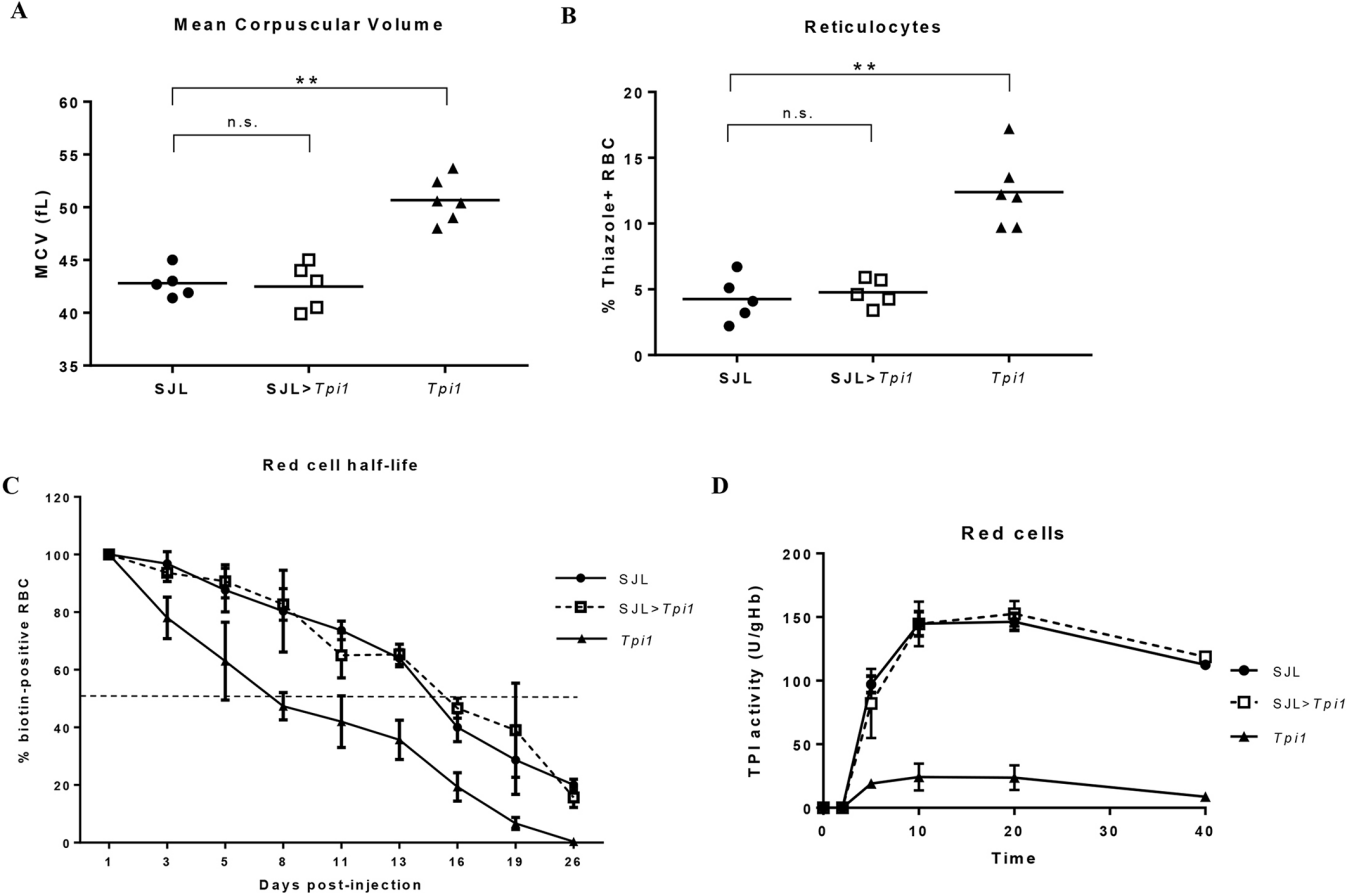
**Bone marrow transplantation corrects macrocytosis and haemolysis.** (A,B) Red blood cell mean corpuscular volume (MCV) (A) and circulating reticulocytes (B), measured in the peripheral blood of 7-week post-transplanted mice. Individual values are shown, along with the mean (solid line). (C) Red blood cell half-life measured *in vivo* in 7-week post-transplanted mice using biotinylation, demonstrating the increased half-life of circulating red blood cells in the transplanted (SJL>*Tpi1*) cohort; *n*=4. Error bars represent s.d. (D) Kinetic assay showing TPI enzyme activity, measured in the peripheral red blood cells of 7-week post-transplanted mice over a time-course of 40 min. Enzyme activity within the red blood cells of the transplanted cohort (SJL>*Tpi1*) shows equal catalytic function to the wild-type enzyme; *n*=3. Error bars represent s.d. **P<0.001, n.s.: not significant.

To determine whether the erythroid phenotype in *Tpi1^F57S/F57S^* mice could be rescued, bone marrow transplants were performed using wild-type (SJL) mice. *Tpi1^F57S/F57S^* recipients, reconstituted with wild-type bone marrow, showed restoration of all peripheral red blood cell parameters after 7 weeks’ recovery, including reduced MCV (Fig. 4A) and reduced reticulocytes (Fig. 4B), with levels comparable to those of the wild-type control cohort (SJL). The *in vivo* red blood cell half-life, measured by biotinylation, was identical to that of wild-type red blood cells (Fig. 4C). In addition, TPI enzyme activity within peripheral red blood cells had returned to 100% of wild-type activity levels (Fig. 4D). In contrast, *Tpi1^F57S/F57S^* mice reconstituted with *Tpi1^F57S/F57S^* bone marrow showed persistent macrocytosis, shortened red blood cell half-life, and reduced red blood cell TPI activity at 7 weeks (Fig. 4A-D). Thus, the macrocytic haemolytic anaemia in the *Tpi1^F57S/F57S^* mutant strain, being intrinsic to haematopoiesis, could be rescued using bone marrow transplantation.

## DISCUSSION

In the present study, we have described a novel ENU mutagenesis mouse strain harbouring a homozygous loss-of-function mutation in the *Tpi1* gene, resulting in a macrocytic haemolytic phenotype that closely resembles human TPI deficiency. A phenylalanine-to-serine substitution at amino acid 57 severely reduced enzyme activity in multiple tissues, particularly those with a high dependence on anaerobic glycolysis, such as red blood cells, bone marrow and neurons. This novel model of TPI deficiency was utilised to demonstrate that the haematological phenotype could be rescued by transplantation using wild-type donor bone marrow. Transplantation successfully eliminated macrocytosis and haemolytic anaemia, improved the *in vivo* red blood cell lifespan, and increased red blood cell TPI enzyme activity to wild-type levels. This study is the first to recognise that the haematological defects associated with TPI deficiency can be rescued by bone marrow transplantation.

The glycolytic housekeeping protein, TPI, is a ubiquitously expressed enzyme responsible for maintaining equilibrium of the metabolic intermediates DHAP and GAP (Ralser et al., 2008; Orosz et al., 2009; Sarper et al., 2013). Homozygous or compound heterozygous mutations occurring in the *TPI1* gene cause the rare enzymopathy TPI deficiency. Approximately 13 mutations spanning less than 50 reported cases of the disease have been identified in humans so far (Schneider, 2000), each demonstrating variable degrees of enzyme deficiency, anaemia and neurodegeneration (Fermo et al., 2010; Hollán et al., 1993). Nonspherical haemolytic anaemia is a hallmark of TPI deficiency, often identified at birth, and has a chronic effect on patient health (Sarper et al., 2013; Clay et al., 1982). No treatment options have been established for TPI deficiency to date, and no incidence of transplantation or similar trial has been recorded in humans or a mammalian model of the disease. Patients rarely survive beyond childhood, prompting the need to generate more effective long-term clinical management strategies for this devastating disease.

The haematological phenotype identified in the ENU-generated RBC19 (*Tpi1^F57S/F57S^*) mouse strain closely matched the human condition, presenting with a haemolytic phenotype, nonspherical red blood cells, and evidence of erythropoietic stress on extramedullary organs. Despite a slightly elevated MCV and peripheral reticulocyte numbers, heterozygous *Tpi1^+/F57S^* mice did not display the characteristics of haemolytic anaemia and were therefore excluded from most tests. This heterozygous population resembled the silent carriers frequently described in human cases, typically associated with a residual enzyme activity of >50% in all cells (Schneider, 2000). Otherwise, the degree of residual TPI enzyme function observed in the erythrocytes of homozygotes fell within the range reported in human case studies (Valentin et al., 2000; Hollán et al., 1993). Enzyme activity of peripheral blood cells is the gold standard for identifying TPI deficiency. The variation in enzyme function observed in other tissues was likely related to the rate of protein synthesis occurring in those cells. Ultimately, bone marrow transplantation of wild-type donor cells eliminated the macrocytic haemolytic phenotype in *Tpi1^F57S/F57S^* recipients within 7 weeks. The restored red blood cell half-life and peripheral red blood cell TPI activity were key indicators of the transplant's effectiveness, the results of which might prove to be translatable to the treatment of TPI deficiency in humans.

The neurological symptoms often associated with TPI deficiency vary in onset and severity, but do not appear to have a direct correlation to enzyme activity (Ralser et al., 2008; Ahmed et al., 2003). A genotype-phenotype relationship has been suggested, as specific mutant alleles, particularly those that disrupt TPI protein dimerisation, have been shown to correlate with more severe neurodegenerative phenotypes in patients (Orosz et al., 2006; Roland et al., 2016). Compounding genetic factors affecting other metabolic pathways, particularly glyoxal pathways, have also been suggested to play a role in the accumulation of potentially neurotoxic metabolic intermediates, such as DHAP or methylglyoxal (Ahmed et al., 2003; Oláh et al., 2005). On a genetic level, the *Tpi1^F57S^* mutation does not represent a known variant observed in humans. Additionally, 3D protein modelling revealed that the mutational site is unlikely to directly impact protein dimerisation, based on its position in the context of TPI's well-characterised crystal structure (Mainfroid et al., 1996). Physiologically, the mutant mouse does not show any loss of motor function with age, nor any histological evidence of neuropathy. Taken together, we conclude that the *Tpi1^F57S/F57S^* mouse strain presents exclusively as a model of the haematological defects associated with TPI deficiency.

This study is an example of the power of forward-genetics screens in the development of animal models that accurately recapitulate rare human diseases, and subsequently the use of those models in trialling new therapeutic options with clinical translatability. The ENU-generated *Tpi1^F57S/F57S^* mouse strain, a novel model of TPI deficiency, was found to harbour the key haematological characteristics of the human disease, which are targetable by bone marrow transplantation. Our studies of this model hopefully bridge a significant gap in the clinical management and treatment of this rare haemolytic enzymopathy.

## MATERIALS AND METHODS

### Mice

A G1 pedigree (RBC19; *Tpi1^F57S^*) displaying macrocytosis was identified in an ENU mutagenesis screen on an SJL background, as described previously (Brown et al., 2013; Bauer et al., 2015). Mice were genotyped by PCR amplification of the region spanning the F57S mutation using cDNA and the primers: Forward (5′-AGAGAGCCGTGCGTTTGTA-3′) and Reverse (5′-GCCCCATTGGTCACTTTGTA-3′); followed by sequencing using the Big Dye Terminator^TM^ reagents (Australian Genome Research Facility) to identify the point mutation. All animal experiments were approved by Monash University and the Animal Ethics Committee (AEC) of the Alfred Medical and Research Education Precinct. Animals were used and cared for in accordance with AEC guidelines.

### Next-generation sequencing

Massively parallel sequencing (whole exome) was performed on the Illumina HiSeq platform (Australian Genome Research Facility) using the genomic DNA of G_2_ homozygous mice, determined by high MCV. Reads were assembled against the SJL reference sequence, which identified a point mutation at position c170 of *Tpi1* (NC_000072.6), resulting in a phenylalanine to serine substitution at amino acid 57 (F57S).

### Computer modelling

The 3D structure of wild-type TPI1 (PBD entry 4POC) was modified to introduce the F57S substitution using Coot (Emsley et al., 2010). The two-dimensional (2D) contact map for the wild-type and F57S residue was generated using LogiPlot+ (Laskowski and Swindells, 2011). Structures were inspected and figures were generated using PyMOL (version 2.0.3).

### Enzyme assays

TPI enzyme activity was measured colorimetrically using a kit from BioVision (Milpitas, CA, USA). For red blood cell measurements, EDTA-treated blood samples were centrifuged at 4000 ***g*** for 15 min and the packed red blood cell volume was diluted in dH_2_O to a 1:20 dilution, followed by three freeze-thaw cycles with intermittent vortexing to ensure complete lysis. Other tissues were collected in PBS (pH 7.0-7.4) and separated using a 40-μm cell strainer (Stem Cell). Lysates were made in TPI enzyme kit diluent containing a protease inhibitor cocktail (‘cOmplete’, Sigma-Aldrich, MO, USA) followed by three freeze-thaw cycles. Prior to testing, all cell lysates were centrifuged at 4000 ***g*** for 5 min to allow collection of the supernatant. Activity was measured using a Multiskan^TM^ microplate photometer (Thermo Fisher Scientific, Scoresby, Australia) in kinetic mode at 37°C for a maximum of 40 min.

### Serum assays

Bilirubin and lactate were measured colorimetrically in 96-well plates using assay kits obtained from Sigma-Aldrich, and absorbance was read on the Multiskan^TM^ microplate photometer (Thermo Fisher Scientific). DHAP was measured fluorometrically using a PicoProbe^TM^ assay kit (BioVision), and analysed on a FLUOstar OPTIMA (BMG Labtech, Victoria, Australia) on fluorescent intensity mode with an excitation wavelength of 544 nm and an emission wavelength of 590 nm. HbA1c was measured by enzyme-linked immunosorbent assay (ELISA) (MyBioSource, San Diego, CA, USA), and absorbance was read on the Multiskan^TM^ microplate photometer.

### Statistical analysis

Where applicable, results are expressed as mean±s.d. For statistical analysis, a two-tailed Student's *t*-test was employed, unless stated otherwise. *P*<0.05 was considered statistically significant.
